# The effect of chronic inflammation on female fertility

**DOI:** 10.1530/REP-24-0197

**Published:** 2025-03-03

**Authors:** Stephen Ameho, Michael Klutstein

**Affiliations:** Institute of Biomedical and Oral Research, Faculty of Dental Medicine, The Hebrew University of Jerusalem, Jerusalem, Israel

**Keywords:** fertility, gametes, oocytes, reproductive aging, chronic inflammation, immune cells, interventions, inflammaging

## Abstract

**In brief:**

Chronic inflammation causes serious medical conditions in many organs and tissues, including female fertility. Here we review the current literature, showing that chronic inflammation has a negative impact on oocyte quality, folliculogenesis, hormone production, immune signaling and other processes that affect fertility in females.

**Abstract:**

Inflammation has key biological roles in the battle against pathogens and additional key processes in development and tissue homeostasis. However, when inflammation becomes chronic, it can become a serious medical concern. Chronic inflammation has been shown to contribute to the etiology and symptoms of serious medical conditions such as ulcerative colitis, cardiovascular diseases, endometriosis and various cancers. One of the less recognized symptoms associated with chronic inflammation is its effect on reproduction, specifically on female fertility. Here we review the current literature, showing that chronic inflammation has a negative impact on oocyte quality, folliculogenesis, hormone production, immune signaling and other processes that affect fertility in females. We discuss several factors involved in the etiology of chronic inflammation and its effect on female fertility. We also discuss possible mechanisms by which these effects may be mediated and how interventions may mitigate the effect of chronic inflammation. Finally, we discuss the notion that in many cases, the effect of chronic inflammation is tightly correlated with and resembles the effect of aging, drawing interesting parallels between these processes, possibly through the effect of aging-associated inflammaging.

## Introduction

The immune system has many diverse roles in development and homeostasis, as well as in the battle against infection. In the state of infection or injury, the body elicits an immune response by recruiting immune cells to fight off the infection or repair the injury. There are two main phases of inflammation, termed acute and chronic inflammation. In the acute phase of inflammation, damaged tissues and foreign bodies are swiftly healed and destroyed, respectively, with symptoms lasting for a few days to a few weeks (Pahwa *et al.* 2024). Usually, acute inflammation is resolved once the infection has been eradicated. However, in cases where the source of infection cannot be eradicated or where, for other reasons, there are repeated cycles of acute inflammation—chronic inflammation may emerge. Chronic inflammation may last for months to years.

Chronic inflammation is a serious medical condition that damages tissues and cells around the body. Recently, chronic inflammation has been identified as a leading cause of death, amounting to about 50% of the global population in cases of neurodegenerative diseases, autoimmune diseases, cardiovascular diseases and cancer. (Abay *et al.* 2018). Chronic inflammation is generally characterized by the colonization of infected or injured sites with monocytes, macrophages and lymphocytes, along with the proliferation of connective tissues and blood vessels. These immune cells produce enzymes and cytokines that can cause long-term damage to cells, tissues and organs. The main systemic features of chronic inflammation include susceptibility to infections, gastrointestinal disorders (e.g. acid reflux, constipation and diarrhea), weight loss or gain, insomnia, prolonged tiredness, arthralgia, body pain, myalgia, anxiety, depression and mood disorders (Pahwa *et al.* 2024). The duration and resolution of chronic inflammation are heavily influenced by external factors or internal processes. Among others, external factors include lifestyle (diet and smoking), environmental pollutants and genetics. Chronic inflammation can also be acquired from several autoimmune diseases such as systemic lupus erythematosus (SLE) and type I diabetes. While acute inflammation is a critical and typically temporary response that promotes healing, the persistence of inflammation over time can lead to the development of chronic inflammation, a condition with far-reaching and severe consequences for the body.

Among the different processes in a body affected by chronic inflammation, one of the least studied is female reproduction and fertility. Chronic inflammation affects female fertility in diverse ways through the disease conditions that are generated because of inflammatory processes. For example, during endometriosis, large ovarian masses are formed, adhesions are generated and the germinal epithelium may be damaged due to inflammation, resulting in an alteration of the pelvic anatomy, tubal obstruction and a reduction in the number of oocytes produced in the ovaries (Weiss *et al.* 2009). In another important example, primary ovarian insufficiency (POI) is a state in females characterized by a major decline in follicle number and ovarian function between puberty and 40 years. Even though POI can be caused by genetic defects such as galactosemia, it can also be acquired from external sources such as iatrogenic origins (chemotherapy, surgery and radiation), infections (e.g. cytomegalovirus), mutations (e.g. mutations in luteinizing hormone (LH) receptor), autoimmune diseases (e.g. polyglandular autoimmune syndrome) and lifestyle (e.g. smoking) (Beck-Peccoz & Persani 2006, Weiss *et al.* 2009). Among these causes, many determinants involved in POI have a strong connection with chronic inflammation. Thus, it is evident that many cases of infertility in females have an element of chronic inflammation directly or indirectly linked to them. Building on the foundational understanding of inflammation and its impact on health and reproduction, it is essential to explore the central mechanisms and key players of inflammation, which orchestrate both protective and pathological immune responses in the body. Below, we discuss how inflammatory pathways can affect female fertility and what can be done to mitigate these effects.

## Central roles of inflammation

The immune system functions to differentiate between self or foreign antigens, injury and healthy cells of the body. It does so by maintaining healthy cells, distinguishing between self and foreign antigens, and then eliminating the foreign ones while healing injuries. During the process of identifying and eliminating foreign bodies and healing injuries, immune cells are produced in the bone marrow and migrate to peripheral sites such as the thymus, spleen and lymph nodes, where they mature and reside until needed for an immune response.

Many immune cells participate in the inflammatory process, mainly through direct action such as the secretion of antibodies, phagocytosis or the production and secretion of cytokines that affect other cells. Such cytokines include factors such as tumor necrosis factor-alpha (TNF-α), IL-6, interferon-gamma (IFN-γ) and IL-1b. For example, in the case of injury, these immune cells stimulate endothelial cells at the site to produce integrins and selectins, which in turn activate diapedesis and chemotaxis of the white blood cells (WBCs, i.e. leukocytes). Dendritic cells and macrophages help in phagocytizing antigens, presenting antigens to lymphocytes, and helping in the release of cytokines. When leucocytes get to the site of injury, they become activated by dendritic cells and macrophages, resulting in the release of inflammatory mediators and cytokines (Pahwa *et al.* 2024). Thereafter, platelets are stimulated and tend to secrete soluble factors and interact with other immune cells, which aid in promoting physical reactions of inflammation such as scars. Platelets are actively involved in tissue damage and the restoration of damaged tissues, resulting in tissue fibrosis (Scherlinger *et al.* 2023). Neutrophils are another group of WBCs that act as major players at the onset of inflammation. They play a role in phagocytosis and destroy foreign antigens by secreting matrix metalloproteinases, reactive oxygen species (ROS), cytokines, lysosomes and myeloperoxidase. Lymphocytes (B and T lymphocytes) also mediate inflammatory responses through complex processes by producing cytokines and their co-activation, secretion of antibodies and antigen–antibody complex (Pahwa *et al.* 2024). Classically, inflammation can be characterized based on four fundamental signs, including pain, redness, swelling and heat. Blood vessels become inflamed with increased activity of macrophages (neutrophilic leukocytes) and macrophages (monocytes). In addition, histamines are released to induce vascular changes, while the complement mediates the antibacterial effect (Pahwa *et al.* 2024). Inflammation is often associated with its central role in immune defense and tissue repair, but its processes and mediators also extend beyond injury response to play critical and multifaceted roles in the regulation of physiological systems, including the intricacies of female reproduction.

## Benefits of inflammatory processes and immune cells in female reproduction

Players of the immune system have a vital role in maintaining the physiological state of the female reproductive system (Dai *et al.* 2023). As a result of the female reproductive tract’s (FRT’s) capacity to elicit a wide range of immune responses, the immune system has evolved to meet challenging physiological demands in terms of protection against microbial attacks and successful reproduction (Nguyen *et al.* 2014). In the FRT, macrophages, dendritic cells, T cells, NK cells and neutrophils dynamically regulate the immune microenvironment (Dai *et al.* 2023). In the upper FRT (the oviduct, the womb and the endocervix), these cells establish immunological tolerance for sperm and embryos while maintaining protection against infections in the lower reproductive tract (vaginal tract and exocervix). These cells are also crucial for trophoblast migration, decidual angiogenesis and immunological tolerance during pregnancy (Lee *et al.* 2015). Immune cells influence the development, regression and modulation of luteotropic and luteolytic processes in the corpus luteum (Walusimbi & Pate 2013). Hence, infertility and pregnancy-related problems are linked to the dysregulation of endometrial/decidual immune cells (Lee *et al.* 2015).

Macrophages (Mφ) emerge as key players within the ovary, demonstrating remarkable functional versatility in supporting both immune defense and the regulation of reproductive processes such as folliculogenesis, ovulation and implantation. Macrophages are dominant cells that can differentiate into M1 (pro-inflammatory) and M2 (anti-inflammatory) and play the roles in the elimination of cellular pathogens and tissue repair, remodeling and reduction of inflammation, respectively (Dai *et al.* 2023). M1 macrophages have also been shown to play a crucial role in several processes of normal ovarian function and female fertility, in terms of folliculogenesis, ovulation count and rates of fertilization and implantation (Ono *et al.* 2018). Clusters of macrophages found in pre-evolutionary follicles have been shown to play crucial roles in ovulation processes. Apart from the pro- and anti-inflammatory responses, macrophages (Mφ2, 3 and 4 clusters) produce exosomes that function in remodeling the extracellular matrix (ECM), while Mφ5 can be linked with chemotaxis and granulocytes. Macrophages also interact intimately with dendritic cells, T cells and neutrophils (Wu *et al.* 2022). This symbolizes the role of macrophages in recruiting immune cells to maintain self and environmentally harmless antigens while fighting against invasive pathogens in the female reproductive system. The immune milieu of the female follicle fluid contains cells such as macrophages, IL-6, IL-12, dendritic cells, natural killer cells and soluble HLA-G and an imbalance or a slight shift in these cytokines and immune cells can constitute a variation in follicle development, oocyte quality and maturation and ovulation (Fainaru *et al.* 2012, Dai *et al.* 2023). Similarly, the signaling cascades involved in ovulation have several mediators that are common in inflammatory processes. During ovulation, there is a surge in LH, which initially generates a response in theca and granulosa cells to produce inflammatory mediators such as cytokines, chemokines, steroids and prostaglandins. These mediators, in turn, regulate pathways that modify the structure of follicles, including the alteration of the follicle stroma, disruption of the basal lamina of granulosa cells, initiation of cumulus expansion and detachment of cumulus–oocyte complexes and also permit the invasion of vascular cells (Duffy *et al.* 2019).

It has been shown in several cases that inflammation caused by certain immune cells is needed for female reproductive processes. For example, during implantation and the early stages of pregnancy, chemokines activate CD4+ T cells, which are deployed to the maternofetal interface to mediate the formation and maintenance of a balanced immune tolerance of the interface (Logiodice *et al.* 2019). Hence, an in-depth knowledge of how immune cells influence reproduction in females can be a major step toward improving female fertility and developing novel techniques and treatments for conditions related to infertility in females.

## Inflammation as a double-edged sword in general health and fertility

Inflammation can be described as a double-edged sword because it protects the body against infection, maintains homeostasis and repairs tissue damage. However, inflammation can damage cells and tissues and can cause autoimmune disease when it becomes chronic and then attacks self-antigens. Because of the vast importance of chronic inflammation in pathogenesis, it has become a focal point of investigation among researchers to advance knowledge and new strategies for effective treatment.

There are various factors that participate in the etiology of chronic inflammation. Here, we will discuss some notable examples: environmental factors, lifestyle factors, obesity and microbial factors. In addition, age can significantly contribute to the appearance and progression of chronic inflammation through a mechanism termed inflammaging. Aging and its connections with chronic inflammation will be discussed at the end of this review.

Besides the effects of chronic inflammation on general aspects of human health, there has been a rising interest in the specific effects of chronic inflammation on female fertility. There are several mechanisms by which chronic inflammation may impact female fertility, which are discussed below. In general, fertility in females is controlled by many factors such as hormone levels, cell differentiation and migration and specific gene functions. Due to the complexity of the processes involved, an imbalance in a single factor can result in a reduction or complete loss of fertility. Chronic inflammation has been identified as one of the factors responsible for various reproductive disorders such as POI and polycystic ovary syndrome (PCOS). A proper understanding of the mechanisms underlying the link between female fertility and chronic inflammation is imperative to developing interventions.

The role of immune cells and cytokines in follicle atresia: immune cells and cytokines also significantly influence follicle atresia, with macrophages and T lymphocytes contributing to granulosa cell proliferation and differentiation through cytokines such as GM-CSF, TNF-α and IL-6. In follicle atresia, these immune cells and cytotoxic T lymphocytes play a pivotal role in regulating the degeneration of non-dominant follicles by inducing granulosa cell apoptosis and clearing cellular debris through phagocytosis. Neutrophils and mast cells assist in ovulation by promoting inflammation and follicular rupture, while regulatory T lymphocytes modulate inflammation to maintain corpus luteum function via IL-10 and TGF-β. Cytokines such as TNF-α and IL-1β are central to triggering apoptosis, promoting immune cell recruitment, and facilitating the degradation of atretic follicles through inflammatory mediators. This coordinated immune response ensures the removal of degenerated follicles, maintaining ovarian homeostasis, and preventing disruptions that could impact fertility (Alesi *et al.* 2021).

In parallel to the factors accountable for chronic inflammation mentioned above, all these factors have also been linked to female fertility.

### Environmental exposures

In the modern era, humans are exposed to inevitable dangers posed by the environment due to changes introduced through the industrial revolution that lead to inflammatory challenges. Technological advances have also contributed to changes that affect humans and consequently increase the evolution of new diets, psycho-emotional stress and information overload. The combination of these factors eventually constitutes a danger signal in the environment, which constantly activates the central stress axes in the body and the innate immune system, mainly through exposure to specific chemicals (Bosma-den Boer *et al.* 2012, Kharrazian 2021, Shetty *et al.* 2023).

Several studies have found significant correlations between the level of ovarian reserve (anti-Müllerian hormone (AMH)) and the levels of air pollution (Abareshi *et al.* 2020, La Marca *et al.* 2020, Kim *et al.* 2021, Bongaerts *et al.* 2023, Pang *et al.* 2023). Moreover, a recent study has directly found the presence of black carbon particles in ovarian sections from consenting gender reassignment patients, showing that air pollution can directly reach ovarian tissue (Bongaerts *et al.* 2023).

The effects of environmental exposure on fertility have been investigated in the last decades, with many studies showing direct negative effects of environmental pollutants on female fertility in cellular and animal models. For example, a recent study found that exposure of human ovarian tissue *in vitro* to phthalates reduced follicular growth and increased follicular degeneration in the tissue (Panagiotou *et al.* 2024). Another study found that phthalate exposure in mice disrupts the ovarian IGF1 system, thus reducing ovarian reserve (Jauregui *et al.* 2023). Another study showed that phthalates cause epigenetic changes in mouse primordial follicles, reducing the ability of the oocyte to modify histone with H3K4me3, an activating modification that promotes transcription of key genes in folliculogenesis (Li *et al.* 2023*a*). More notable examples are detailed below.

Components of PM_2.5_ (fine inhalable particles with an aerodynamic diameter of ≤2.5 μm) have been identified to cause inflammatory diseases with varying toxicity. Among the many constituents, it was shown that airborne metals associated with steel generated the highest level of inflammatory response (Ghio & Devlin 2001, Franklin *et al.* 2008, Bell *et al.* 2014, Kumarathasan *et al.* 2018, Tripathy *et al.* 2021). Genetic influence in the response to these pollutants can potentially account for part of the causes of chronic inflammation, but it seems the combinatorial effects of environmental factors with either causative or amplifying roles contribute about 75% of inflammatory response in victims (Bachmann *et al.* 2020). Importantly, the influence of these particles was shown to be important for female health. Significant levels of inflammatory cytokines such as IL-6, TNF-α and IL-1b were found in individuals who had long-term exposure to PM_2.5_, which included black carbon, lead, manganese, zinc and iron. The results show that the relationship between the stimulated cytokines and the heavy metals was consistently higher in females compared to their male counterparts, but the difference was only significant for IL-6 (Tripathy *et al.* 2021).

Bisphenol A (BPA) is one of the most widely produced environmental endocrine disruptors, with over 3 million tons produced per year. BPA is prevalent in the environment due to its extensive use in products such as polycarbonates, epoxy resins and thermal paper, resulting in everyday exposure through food packaging, plastic bottles, water pipes, electronics, paper and toys (Thompson *et al.* 2015). A recent study has highlighted BPA’s complex effects on the immune system, which can activate or inhibit immune responses. This inconsistency suggests that BPA disrupts the balance of the innate immune system rather than simply targeting specific adaptive immune responses. BPA also appears to influence various immune cell types, especially within the T-cell population. Evidence suggests that BPA promotes the proliferation of immune cells (Rogers *et al.* 2013, Thompson *et al.* 2015), but its specific effects on different immune cells are complex and not fully understood. For example, CD4+ T lymphocytes include both pro-inflammatory cells (such as Th1 and Th17) and anti-inflammatory cells (such as Th2 and regulatory T cells). Studies show mixed results regarding BPA’s impact on these cells; some indicate that BPA activates Th1 and Th2 cells, with the balance between these responses varying depending on the dose, timing and duration of exposure. It has been shown that prenatal BPA exposure significantly inhibits Treg cells, raising important questions about how BPA might contribute to susceptibility to T cell-mediated inflammation (TAI) (Yan *et al.* 2008). The exact reasons for the polarization of BPA-exposed CD4+ T cells toward either a pro-inflammatory or anti-inflammatory state remain unclear. However, evidence indicates that BPA affects CD4+ T cells at exposure levels similar to those experienced by humans. In addition, studies on macrophages and B cells show conflicting results regarding BPA’s impact on these immune components as well (Rogers *et al.* 2013). Overall, the potential effects of BPA on inflammation and female fertility highlight the need for further research, especially considering its widespread use and associated health risks.

Polybrominated diphenyl ethers (PBDEs) are another category of environmental agents that are flame retardants, and they are found in many everyday products, from furniture to electronics. These chemicals can leach from products, contaminating air, food, water and soil. PBDEs tend to accumulate in human fat tissue, where they can persist for 1–3 years. Over the last few decades, levels of PBDEs in the human body have risen, with significant concentrations detected in breast milk, placentas and umbilical cord blood, particularly in people living near electronic waste sites (Thompson *et al.* 2015). Although PBDEs aren’t direct mutagens, they can interfere with immune responses. For example, studies on placental tissue exposed to PBDEs revealed that the presence of certain bacteria switched the immune response from anti-inflammatory to pro-inflammatory, triggering cytokines such as IL-1β and TNF-α, which drive inflammation. This suggests that PBDEs may make the body more reactive to bacterial infections, potentially intensifying inflammation (Thompson *et al.* 2015). This immune disruption could have broader health impacts. Chronic inflammation is known to raise cancer risk, especially for cancers linked to bacterial infections, such as stomach cancer associated with *H. pylori*. PBDEs may promote low-grade, chronic inflammation, disrupting the body’s balance with its normal microbiota. This disturbance could create an inflammatory environment in the female reproductive system, potentially leading to infertility or reproductive defects.

Phthalates are a group of commonly used plasticizers derived from phthalic acid and they constitute a significant health concern due to their widespread presence in our environment and their multiple pathways of exposure. We encounter these chemicals through food and drink, inhalation, skin contact and even medical procedures such as blood transfusions. For example, diethylhexyl phthalate (DEHP) is often found in plastic food packaging, especially when heated in the microwave, which can lead to contamination. Most health concerns regarding phthalates have focused on reproductive health, particularly how they affect sperm production. Early-life exposure is particularly alarming, as phthalates are recognized as weak anti-androgens that can disrupt testosterone synthesis in Leydig cells (Thompson *et al.* 2015). Moreover, high levels of phthalates have been detected in children from toys and personal care products, raising additional concerns (Tsai *et al.* 2012). Various studies suggest that phthalates can act as immune disruptors. Although *in vitro* and *in vivo* studies indicate that phthalates can affect immune cells such as macrophages, lymphocytes, eosinophils and neutrophils, the results have been inconsistent and often depend on the context. For instance, chronic exposure to airborne DEHP increased the number of eosinophils and lymphocytes in lung lavage fluid, but only at very high concentrations that do not reflect typical human exposure. On the other hand, low doses of MEHP, a major metabolite of DEHP, exhibited pro-inflammatory effects, underscoring how metabolic processes can influence the impact of these chemicals (Larsen *et al.* 2007). Interestingly, a study involving allergic individuals found that low DEHP exposure led to increased levels of pro-inflammatory markers, such as granulocyte colony-stimulating factor and IL-5, while higher exposure resulted in a suppression of these same markers (Deutschle *et al.* 2008). This suggests that phthalates can have both inflammatory and immunomodulatory effects, depending on the dose, duration of exposure and tissue type involved. These complex interactions make it challenging to directly apply findings from animal and lab studies to human health outcomes, but they highlight the potential risks associated with phthalate exposure. The immune-disrupting nature of phthalates is further confirmed by findings from the Comparative Toxicogenomics Database, which indicates that phthalates disrupt several toxicity networks related to inflammation, affecting tissues commonly associated with cancer, such as the prostate, uterus, ovaries and breast (Singh & Li 2012). Notably, phthalates demonstrate gene expression disruptions that closely resemble those seen with other endocrine disruptors such as BPA. This suggests a shared mechanism affecting inflammatory responses, which could prove valuable for future research into the inflammatory potential of various environmental chemicals.

Vinclozolin is an environmental toxin (dicarboximide fungicide) that has been used for several decades. While the World Health Organization (WHO) considers it unlikely to pose an acute hazard in normal usage due to its low toxicity in rats, the Environmental Protection Agency (EPA) raises significant alarms. They report that Vinclozolin, along with its breakdown product 3,5-dichloroaniline, can induce testicular tumors in rats and tumors in the kidneys and prostate of dogs. Consequently, the EPA has classified Vinclozolin as a possible human carcinogen, a designation not shared by the International Agency for Research on Cancer (IARC) or the National Toxicology Program (NTP) (Thompson *et al.* 2015). More compelling than the potential cancer risks are the concerns surrounding Vinclozolin’s effects as an endocrine disruptor. Research indicates that it can lead to anti-androgenic effects, impacting lipid metabolism and storage, lower sperm counts, decreased prostate weight and delayed puberty in animal studies (Rider *et al.* 2010). A critical area of concern is Vinclozolin’s link to inflammation, particularly following early-life exposure. Studies have shown that transient exposure to Vinclozolin during pregnancy can lead to inflammation-related diseases later in life (Skinner & Anway 2007, Anway & Skinner 2008). For instance, in post-pubertal rats, exposure *in utero* has been linked to prostatitis, characterized by a decrease in androgen receptor (AR) activity and an increase in nuclear NF-κB levels. This suggests that exposure to Vinclozolin during key developmental windows may result in permanent changes in DNA methyltransferase activity, which reprograms AR genes and manifests as inflammation in adulthood, negatively affecting spermatid numbers. Moreover, the evidence points to the possibility of transgenerational effects, with findings showing that defects in spermatogenic cells can persist into the third generation (F3) (Skinner & Anway 2007, Thompson *et al.* 2015). This suggests that specific changes in gene methylation can be inherited, linking environmental exposures to long-term health issues. These insights underscore the need for more comprehensive research into Vinclozolin’s molecular mechanisms and its role in inflammation-related diseases and risks associated with female reproduction.

4-Nonylphenol (4-NP) is another pervasive environmental chemical that has recently been implicated in promoting inflammation. People can be exposed to 4-NP through contaminated food and water, primarily from everyday products such as liquid detergents, cosmetics, paints and pesticides that utilize nonylphenol ethoxylates as nonionic surfactants (Careghini *et al.* 2015). Interestingly, the concentrations of 4-NP are often higher in treated wastewater than in the sources due to microbial breakdown of the parent compound, nonylphenol ethoxylate (Pasquini *et al.* 2014). Known for its significant reproductive effects, 4-NP acts as an endocrine disruptor, raising concerns about its impact on female fertility. Research indicates that prenatal exposure to 4-NP can result in increased levels of progenitor white adipose tissue, as well as higher body weight and overall size in rodents. Similar to Vinclozolin, these changes in fat development during crucial developmental periods can lead to transgenerational inheritance of obesity-related traits in the F2 generation, suggesting an epigenetic reprogramming process (Zhang *et al.* 2014). The pro-adipogenic effects of 4-NP are also associated with a decrease in estrogen receptor alpha (ERα) levels in adipose tissue, reflecting its weak endocrine-disrupting capabilities, alongside the activation of genes involved in fatty acid metabolism and fat storage, including *Ppar-γ*, *Srebp-1*, *Lpl* and *Fas*. Furthermore, recent research has shed light on 4-NP’s potential role as an immune disruptor. Studies found that exposure to 4-NP increased the expression of cyclooxygenase-2 (COX-2) in a mouse macrophage cell line (RAW264.7), leading to a significant rise in prostaglandin E2 (PGE2) production. This shows that 4-NP activates signaling pathways involving Akt and MAP kinases, which are crucial for COX-2 expression (Thompson *et al.* 2015).

Atrazine, one of the most widely used herbicides globally, is frequently detected in waterways due to its heavy use in agriculture to control broadleaf weeds and grasses (Hayes *et al.* 2010). Recent research links atrazine to various health concerns, especially regarding its role as an endocrine disruptor and its impacts on reproductive health. Studies have shown that atrazine can lead to mammary tumors in rodents (Cooper *et al.* 2007) and disrupt male reproductive systems (Stanko *et al.* 2010). Findings by Jin *et al.* suggest that both atrazine and its main metabolite, Diaminochlorotriazine, can weaken the liver’s antioxidant defenses and reduce gene expression tied to testosterone production, indicating that oxidative stress may underlie some of these reproductive effects (Jin *et al.* 2014). Atrazine also impacts nitric oxide (NO) production, an essential molecule for various bodily functions, including immune responses and tumor suppression. Research in swine granulosa cells found that atrazine exposure increases both NO and VEGF, potentially promoting blood vessel growth (angiogenesis) associated with tumor development (Thompson *et al.* 2015). In addition, in mouse models, Atrazine exposure showed immunotoxic effects by reducing both cell-mediated and humoral immunity, including lowered NO production by macrophages (cells critical to immune defense and tumor suppression) (Angst *et al.* 2013). Atrazine exposure further decreased key immune-signaling proteins such as TNFα and IFN-γ and inhibited lymphocyte and natural killer cell functions, both crucial in fighting infections and cancer (Thompson *et al.* 2015). These findings raise concerns about Atrazine’s inflammatory and immunosuppressive effects, which could heighten susceptibility to reproductive health issues.

The complex processes by which external exposures disturb homeostasis and reproductive processes are highlighted by the substantial effects that the interaction of environmental factors and chronic inflammation has on both general health and female fertility. Developing focused interventions and reducing the risks that environmental pollutants pose to human health and fertility depend on an understanding of these relationships.

### Lifestyle factors

Studies indicate that modifiable lifestyle factors such as smoking, exercise, diet and sleep play a critical role in both fertility and chronic inflammation. Evidence shows that negative lifestyle factors including smoking and drinking can significantly impact fertility with correlations to ovarian reserve (measured by AMH levels) and implantation rates in IVF after ICSI (Setti *et al.* 2022, Werner *et al.* 2024). In addition, persistent chronic inflammation, driven by lifestyle factors such as poor diet, exercise habits and sleep deprivation, can intensify or promote non-communicable diseases such as obesity, diabetes, infertility and cardiovascular diseases. Here, we delve into the specific impacts of lifestyle factors including diet, exercise and smoking on chronic inflammation and fertility.

Diet may either increase or reduce the progression of chronic inflammation. Diets with high proportions of sodium, saturated and/or trans fats, refined starches and sugar have been reported to contribute to systemic inflammation, in contrast with omega-3 fatty acid, fiber-rich diets and antioxidants, which turn out to be key players in anti-inflammation (Nieman *et al.* 2021). Adipose tissue produces TNF-α, and when its concentrations together with C-reactive protein (CRP) increase in the serum, it can contribute to the development of type-2 diabetes. Different markers of inflammation have also been used to assess the severity of chronic diseases such as diabetes, and the most commonly used markers are high-sensitive CRP (hs-CRP), TNF-α, CRP, IL-1α, IL-1b, IL-1RA, IL-6, IL-8, IL-10, plasminogen activator inhibitor 1 (PAI-1), MIF (macrophage migration inhibitory factor), serum amyloid A (SAA) and RANTES (regulated on activation, normal T-cell expressed and secreted) (Uusitupa & Schwab 2013, Wang *et al.* 2013). Recent studies have associated elevated levels of these inflammatory markers with the consumption of low-fiber diets, excess carbohydrates or diets with a high glycemic index (GI) and diets with high saturated fatty acids (SFAs), and lower levels of omega (ω)-3 fatty acids in the diet, most of which showed significant correlation to hs-CRP, IL-1, CRP, IL-6 and TNF-α (Galland 2010).

Exercise can be considered beneficial or harmful depending on its intensity. Extreme exercise over a long duration can result in the secretion of higher quantities of inflammatory mediators, which may predispose the victim to tissue damage and chronic inflammation. Energetic or moderate exercise with appropriate resting intervals confers therapeutic benefits (Cerqueira *et al.* 2020). Research has shown that there is a variation in the cytokine surge secreted after exercise in correlation with the intensity of exercise (Cabral-Santos *et al.* 2019). The level of IL-10 and TNF-α rises after intense exercise, while IL-1b and IL-6 increase less with moderate exercise compared to intense exercise (Cerqueira *et al.* 2020).

Importantly, exercise has been shown to strongly affect chronic inflammation in females. For example, exercise can reduce CRP levels in women with PCOS (Hafizi Moori *et al.* 2023). Moreover, high-intensity intermittent exercise was shown to induce a strong anti-inflammatory response in healthy women across the menstrual cycle (Minuzzi *et al.* 2022).

Only a few studies investigated the mechanistic links between modifiable lifestyle factors and female fertility or oocyte quality. Focusing on exercise, for example, one study showed that exercise elevates antioxidant levels in the ovary of rats fed a high-fat diet (Furtado *et al.* 2021). Another interesting study showed an improvement in oocyte quality in terms of spindle formation, chromosome segregation and mitochondrial distribution in mtDNA ‘mutator’ mice, also called ‘POLG’ progeroid mice, which have undergone physical exercise treatment—a regimen known to delay their aging (Faraci *et al.* 2018).

Focusing on smoking behavior, some studies reported direct links to molecular changes in the reproductive system. For example, a study showed DNA methylation changes in the granulosa cells of IVF patients who were smokers compared to non-smokers (Tang *et al.* 2024). Exposure of mice to cigarette smoke condensates lowered their reproductive success in both natural mating and IVF (Banafshi *et al.* 2023). Another study examined the effect of mouse oocyte exposure to cotinine—a major metabolite of nicotine and cigarette components. Cotinine exposure caused defects in the first polar body extrusion and reduced parthenogenetic activation in *in vitro*-matured oocytes. In addition, cotinine exposure increased oxidative stress, abnormal spindle morphology, chromosome misalignment and oocyte aneuploidy (Cheng *et al.* 2022).

It is evident that modifiable lifestyle factors such as diet, exercise and smoking have profound impacts on chronic inflammation and fertility, with both beneficial and detrimental effects depending on their nature and intensity. Further research into these mechanisms could provide valuable insights into improving reproductive health and managing chronic conditions.

### Obesity

Obesity and its effect on female fertility are currently one of the most studied topics in this field. Several recent reviews have been written on the subject of the effect of obesity on fertility (Ferrell *et al.* 2023, McLean & Boots 2023, Muhammad *et al.* 2023, Schon *et al.* 2024). It is currently clear that obesity negatively affects female fertility at different levels, including hormone balance, granulosa cell function and oocyte quality.

The adipose tissue serves as an endocrine signaling entity and is responsible for the secretion of several key factors. Adipose-derived cytokines, called adipokines (including leptin, ghrelin, resistin and adiponectin), have important functions in obesity. The secretion of these substances, with more secreted factors related to obesity, leads to hormonal imbalances, low levels of sex hormone-binding proteins, and elevated androgen levels. All these eventually lead to a decrease in both the quantity of mature ovarian follicles and progesterone levels during the menstrual cycle (Wang *et al.* 2008, Itriyeva 2022, Ferrell *et al.* 2023). Studies have shown remarkable differences in granulosa cell transcriptomes in obese mouse females compared to controls (Adamowski *et al.* 2024, Jiang *et al.* 2024). Further studies have shown that obesity induces apoptosis of granulosa cells and changes in their growth and cell cycle patterns (Wu *et al.* 2015, Chen *et al.* 2023). Mouse models have shown that mitochondrial pathway inflammation and oxidative damage are involved in mediating the effect of obesity on oocyte quality (Smits *et al.* 2022, Wang *et al.* 2022, Li *et al.* 2024). All findings in mouse and other animal models have been corroborated in human studies of obese IVF patients, especially those suffering from PCOS (Siemers *et al.* 2023, Anderson 2024, Vatier & Christin-Maitre 2024). Indeed, obesity is known to strongly affect oocyte quality in IVF patients (Jiang *et al.* 2023, Liu *et al.* 2023, Evangelinakis *et al.* 2024).

Clinically, when the body mass index (BMI) of an individual exceeds 30 kg/m^2^ (BMI > 30 kg/m^2^), the individual is considered to be obese (Purnell 2000). An overabundance of fat molecules in the adipose tissues stimulates the production of pro-inflammatory cytokines such as IL-6 and TNF-α, limits the quantity of adiponectin produced by the body, and subjects the body to a state of oxidative stress and chronic inflammation (Ellulu *et al.* 2017). Several inflammatory mediators are produced by fat tissues, including adiponectin, chemokines, resistin, leptin and cytokines such as IL-6, TNF-α and monocyte chemoattractant protein-1 (MCP-1) (Lafontan 2005). According to a study conducted by Straub and colleagues, plasma pro-inflammatory cytokine IL-6 can be linked to parameters of obesity (Straub *et al.* 2000, Hotamisligil 2006). It is suggested that, in the state of hypoxia mediated by the abundance of fat, tissue death and infiltration of macrophages into the fat tissues are encouraged. This causes an upregulation of pro-inflammatory cytokines, which leads to localized inflammation within the fat tissues and, in turn, increases systemic inflammation and elevates the levels of comorbidities associated with obesity (Trayhurn & Wood 2004).

Toll-like receptors (TLRs) are also found in adipocytes, triggered by bacterial lipoproteins and lipopolysaccharide (LPS). TLR2 is attracted to bacterial lipoproteins, while TLR4 also senses LPS. Nuclear factor κB (NF-κB) is translocated to the nucleus upon the interaction of either receptor with its ligand (Wolowczuk *et al.* 2008). It was found that unsaturated fatty acids suppress TLR-mediated signaling and gene expression, while SFAs activate both TLR2 and TLR4 (Qatanani & Lazar 2007). The production of proinflammatory factors such as IL-6, TNF-α and chemokines is triggered by the activation of TLRs. Activation of TLRs in hyperlipidemic situations is expected to occur, leading to increased inflammation (Qatanani & Lazar 2007, Wolowczuk *et al.* 2008).

It is important to mention that obesity is strongly linked to inflammatory phenotypes related to female health. For example, menopause includes a cardiometabolic transition, with many women experiencing weight gain and redistribution of body fat (Barbagallo *et al.* 2024). Another prominent example is the occurrence of PCOS, a multifactorial disease with inflammatory phenotypes, which is highly associated with obesity (Chen *et al.* 2024, Zhang *et al.* 2024).

Thus, it is clear that obesity greatly affects female fertility and can serve as a model case for investigation of the effect of chronic inflammation on fertility.

### Microbial factors

Microbial factors are pivotal causes of chronic inflammation. Inflammation caused by gut microbiota is intended to elicit an immune response against pathogenic strains of microorganisms, aid digestion and prevent cancer. However, in certain cases, microbial-triggered inflammation can induce cancer and tissue fibrosis (Kipanyula *et al.* 2013).

Current evidence suggests that the microbiome has a substantial influence on female fertility and fertility-related syndromes. For example, low levels of estrogen are associated with vaginal dysbiosis, involving the replacement of Lactobacillus in the vagina with anaerobic bacteria such as Gardnerella, Mycoplasma and Prevotella, which increase the vaginal pH to >4.5 (Punzon-Jimenez & Labarta 2021). Another example shows that dysbiosis reduces the gut microbiota and β-glucuronidase activity. This reduction in β-glucuronidase is followed by lower free estrogen reduction in the blood. This is followed by altering estrogen receptor activations, leading to a hypoestrogenic pathologic status such as obesity and PCOS (Dewi *et al.* 2012, Baker *et al.* 2017).

In the state of metabolic endotoxemia (an innate immune response that gradually continues and becomes acute low-grade inflammation due to the circulation of elevated levels of systemic endotoxins), LPSs are created when metabolic and microbial factors interact (Cani *et al.* 2007). Toll-like receptor-4 (TLR-4) is activated, and this upregulates the release of proinflammatory cytokines (Raetz & Whitfield 2002). The main source of endotoxins is the gastrointestinal (GI) microbiota, and the GI microbiota is mostly modulated by diet. The Gram-negative microbiota component of endotoxemia and inflammation is increased by diets high in saturated fat, which also directly activates TLR-4 receptors and increases endotoxemia and inflammation (Lee *et al.* 2001, Cani *et al.* 2007).

Gut microbiota dysbiosis has been associated with the development of several processes of fibrosis of organs. During tissue injury, the body responds by depositing ECM components at the site of injury, and this pathological process is known as fibrosis. Fibrosis is considered the main cause of organ failure and malfunction and a major component of chronic inflammatory diseases (Wynn 2008, Dees *et al.* 2021, Costa *et al.* 2022). Intestinal fibrosis is the most common type of fibrosis, and the process is activated by chronic inflammation-mediated triggering and recruitment of mesenchymal cells (Rieder 2013). The ECM is constantly produced in response to chronic inflammation due to the triggering of mesenchymal cells such as myofibroblasts, fibroblasts and smooth muscle cells. This leads to the possible formation of a barrier in the intestine as a result of structure or fistulae formations (Friedman *et al.* 2013). Upon the immune responses and subsequent deposition of ECM, collagen types I/III are expressed in high levels. This causes further expression of profibrotic mediators, for example, insulin-like growth factor I (IGF-I), transforming growth factor-beta 1 (TGF-β1) and connective-tissue growth factor (Small *et al.* 2013). Bacterial endotoxins also enhance the activation of profibrotic mediators NF-B-light-chain-enhancer of activated B cell (NF-κB) promoter activity and collagen contraction (Burke *et al.* 2010). In the intestinal epithelium, bacterial flagellin activates IL-33 receptor ST2 (as shown in AIEC and an attenuated strain of *S. typhimurium*), increasing IL-33 signaling and the enhancement of intestinal fibrosis (Imai *et al.* 2019, Yang *et al.* 2020*a*). Inflammatory bowel disease (IBD) is a model for studying intestinal fibrosis due to the association between both conditions in terms of microbial-induced inflammation, endothelial wall thickening and deposition of ECM in the mucosal layers. Certain microbes including *Salmonella enterica serovar Typhimurium* and adherent-invasive *Escherichia coli* (AIEC) have been implicated in IBD since they were able to induce inflammation through elevated levels of T-helper (TH) cells in patients or germ-free animal models, resulting in further progression of fibrosis (Grassl *et al.* 2008, Small *et al.* 2013, Ray *et al.* 2014, Imai *et al.* 2019).

Moreover, there are links between pro-inflammatory signals produced in conditions elicited by microbiota such as IBD and female health. For example, IBD can reduce ovarian reserve in women (Guo *et al.* 2023). Another example shows that postmenopausal women with IBD are at higher risk of ischemic stroke than non-IBD postmenopausal women (Greywoode *et al.* 2024).

The main mediators of chronic inflammation in all causative factors described above are summarized in Table 1 (Macarthur *et al.* 2004, Philip *et al.* 2004, Lafontan 2005, Fontana *et al.* 2007, Galland 2010, Patel *et al.* 2013, Uusitupa & Schwab 2013, Wang *et al.* 2013, Viehmann *et al.* 2015, Imai *et al.* 2019, Glencross *et al.* 2020, Yang *et al.* 2020*a*, Nieman *et al.* 2021, Tripathy *et al.* 2021, Michailidou *et al.* 2022, Uribe-Querol & Rosales 2022).

**Table 1:** Major cytokines/chemokines/factors produced in certain inflammatory conditions.

Factors	Major cytokines/chemokines/factors/produced	Common immune cells involved	References
Environmental exposures	IL-1b, IL-6, TNF-α and hs-CRP	Macrophages, neutrophils, eosinophils, dendritic cells, Th2, Th17	Tripathy *et al.* (2021), Viehmann *et al.* (2015), Glencross *et al.* (2020)
Lifestyle factors	IL-6, TNF-α, CRP, hs-CRP, TNF-α, CRP, IL-1α, IL-1b, IL-1RA, IL-6, IL-8, IL-10, PAI-1, MIF and PAI-1	Macrophages, innate lymphoid cells, dendritic cells, neutrophils, eosinophils, mast cells, innate lymphoid cells, natural killer (NK) cells, T- and B lymphocytes	Nieman *et al.* (2021), Uusitupa & Schwab (2013), Wang *et al.* (2013), Galland (2010), Lafontan (2005), Fontana *et al.* (2007), Michailidou *et al.* (2022)
Obesity	IL-6, TNF-α , MCP-1, CRP, TLR-2 and TLR-4	Neutrophils, macrophages, NK cells, T- and B lymphocytes, eosinophils, innate lymphoid cells and mast cells	Michailidou *et al.* (2022), Uribe-Querol & Rosales (2022), Patel *et al.* (2013)
Microbial factors	IL-1b, IL-6, IL-10, TNF-α, IFN, IGF-I, TGF-1, IL-33, TLR-2, TLR-4, NOS and ROS	Macrophages, neutrophils, NK cells, T- and B lymphocytes	Imai *et al.* (2019), Yang *et al.* (2020*a*), Philip *et al.* (2004), Macarthur *et al.* (2004)

## How ovarian function and fertility are impacted by autoimmune disorders

Autoimmune disorders are a major source of chronic inflammation, which can significantly impair ovarian function and fertility in women. Autoimmunity of the ovary, including SLE, POI and polycystic ovariy syndrome (PCOS), involve cell-mediated immune responses (Logiodice *et al.* 2019). It has been shown in humans that autoimmune diseases increase the risk of miscarriage, reduce fecundity and decrease the success of infertility treatments (Gleicher *et al.* 2012). Studies suggest that immune cells can trigger primordial follicle loss via BID-mediated apoptosis in the context of checkpoint inhibitor immunotherapy (Winship *et al.* 2022).

SLE affects women during their reproductive age and predisposes its victims to a variety of ailments. Mild circumstances of SLE may reduce ovarian reserve due to direct effects of the disease on the ovaries, perhaps due to autoimmune oophoritis. Treatment with cyclophosphamide (an alkylating chemotherapy drug known to produce one of the highest gonadotoxic effects on the ovaries of its category) is given to patients with severe SLE, which significantly increases the risk of infertility and early premature ovarian failure. Victims of SLE also experience higher rates of adverse pregnancy outcomes such as preterm birth, miscarriage, growth restrictions in the fetus, preeclampsia and congenital heart defects in newborns while also disrupting the hormonal balance of the hypothalamic–pituitary–ovarian axis, further impairing reproductive function. (Oktem *et al.* 2015).

POI presents reproductive complications often manifesting in a gradual decline of ovarian function that complicates both diagnosis and management, with significant long-term effects on fertility and overall health. Failures caused by POI usually occur gradually and intermittently, complicating both diagnosis and treatment while its systemic effects vary significantly depending on the timing (i.e. whether it occurs before puberty or during reproductive years, as well as the ovarian components that are affected). The underlying conditions that lead to premature ovarian failure often contribute to further systemic symptoms, and in essence, the loss of ovarian function affects many physiological aspects of women’s health beyond just a reduction in estrogen (Taylor 2001). POI is diagnosed when FSH levels are high (over 40 IU/L) and estradiol is low (below 50 pmol/L). POI can be classified as primary (of unknown origin) or as a result of treatments, such as chemotherapy (secondary) (Vujovic *et al.* 2010). POI affects fertility and sexual health, with reduced testosterone levels potentially contributing to sexual dysfunction (Kingsberg 2011). Donor oocyte IVF remains the most effective option for POI individuals who seek to conceive (Vujovic *et al.* 2010).

PCOS is the most prevalent endocrine disorder among women, affecting approximately 5–15% of the population and is a significant contributor to female infertility (Cunha & Povoa 2021). Women with PCOS often face challenges such as irregular menstrual cycles, difficulties with ovulation, higher levels of male hormones (hyperandrogenemia), insulin resistance and accompanied weight loss (Usadi & Legro 2012). These challenges can affect the female reproductive physiology and processes such as oocyte quality, implantation and embryonic development. In PCOS, low levels of progesterone contribute to immune system overstimulation, leading to the production of autoantibodies, which supports its classification as a potential autoimmune disorder. This hypothesis is further supported by documented associations between PCOS and autoimmune conditions, such as the presence of antinuclear antibodies (ANA) seen in SLE and anti-thyroid peroxidase (anti-TPO) antibodies linked to Hashimoto thyroiditis. These findings suggest that autoantibodies in PCOS may have implications for the long-term clinical management of affected patients (Mobeen *et al.* 2016).

Importantly, inflammation is essential in endometrium functions and various reproductive disorders. Despite endometrial dysfunction not being an autoimmune disorder but rather a progressive chronic condition, it is perhaps associated with autoimmune conditions. While the normal cyclical inflammation of the endometrium is a natural process, when it becomes unregulated, it can lead to problems such as heavy menstrual bleeding, painful periods and endometriosis (Maybin *et al.* 2011). Although inflammation is important for healthy endometrial development, tissue repair and remodeling, it can also disrupt females’ reproductive processes, including the endometrium’s ability to support implantation (Weiss *et al.* 2009). In cases of endometriosis, inflammation can cause further complications such as problems with blood vessel function and even an increased risk of cancer (Jiang *et al.* 2016). Interestingly, a controlled inflammatory response is necessary for the uterus to be receptive to a fertilized egg and for successful implantation. Some studies have shown that inflammation from endometrial injury can improve pregnancy outcomes for women who have had multiple failed implantation attempts (Granot *et al.* 2012). This highlights the delicate balance between the beneficial and detrimental effects of inflammation on reproductive health. Understanding this balance is crucial for advancing our knowledge of how inflammation impacts endometrial function and fertility and ultimately helping women achieve better reproductive health outcomes. Hence, these findings underscore the intricate relationship between autoimmune disorders and female reproductive health, highlighting the importance of targeted interventions to safeguard fertility in affected women.

## Possible mechanisms for the effects of chronic inflammation on female fertility

It is becoming clear that chronic inflammation and all the factors associated with it can negatively affect female fertility and can be involved in female reproductive syndromes. However, it is not entirely clear how the signals from remote organs that are under the influence of chronic inflammation (e.g. lungs, gut and even the oral cavity) can reach and affect reproductive organs.

Several hypotheses have been suggested, and support exists for each of them. It should be noted that the hypotheses are not mutually exclusive, and each of them can contribute to part of the symptoms in different syndromes and clinical situations.

### Direct involvement of pathogens

The female reproductive system, especially in its lower parts (the vaginal tract and exocervix), is inhabited by microorganisms that can participate in the initiation of chronic inflammation (Cazzaniga *et al.* 2022). Pathogens can influence reproduction and reproductive organs either by sending signals to organs such as the ovaries or by directly infiltrating these tissues.

For example, specific vaginal pathogens are highly associated with ovarian cancer cases (Shanmughapriya *et al.* 2012, Banerjee *et al.* 2017). One possibility for a connecting mechanism is the depletion of the tissue from iron, thereby starving the resident cells and facilitating damage to these cells (Kawahara *et al.* 2024). Another prominent example is the infiltration of Fusobacterium into the endometrium and its involvement in endometriosis through the increase of endometriotic lesions and phenotypic transition of endometrial fibroblasts (Muraoka *et al.* 2023).

### Disruption of hormonal balance

Chronic inflammation causes various systemic changes along its progression. An interesting and important change during this process is the effect chronic inflammation has on the endocrine system. The secretion as well as the metabolism of several hormones is changed due to inflammation and therefore the steady-state levels of the hormones change in the body. Changes in hormone levels can be a prime source of effects on fertility through multiple mechanisms that are related to the activities carried out by the specific hormone. One example is the lower levels of androgens caused by chronic inflammation. In diseases with a component of chronic inflammation of different etiology, serum androgens are very low (Straub *et al.* 1998, Straub 2014), but estrogen levels remain relatively normal as a consequence of increased conversion of androgens into estrogens in the inflamed tissue (Cutolo *et al.* 2008). Low levels of androgens, especially testosterone, can adversely affect female fertility through the effects of this hormone on folliculogenesis (Hugues & Durnerin 2005, Gleicher *et al.* 2011). Another studied example is the effect of chronic inflammation on energy storage hormones, specifically on insulin. In chronic inflammatory diseases such as rheumatoid arthritis (RA) and SLE, hyperinsulinemia and insulin resistance were described (Dessein & Joffe 2006, El Magadmi *et al.* 2006, Straub 2014). Insulin resistance is a known mediator of female infertility, and this has been demonstrated in many previous studies (Karnatak *et al.* 2022, Vatier *et al.* 2022, Xia *et al.* 2023). Thus, whether through the disruption of sex hormones or through the interaction with other endocrine pathways such as energy storage, chronic inflammation can affect hormonal balance and, through it, female fertility.

### Changes in the ovarian immune milieu

Immune cells constitute part of the normal cell population inside the ovary. Immune cells have important functions in processes that do not relate to defense from pathogens (Zhang *et al.* 2015, Briley *et al.* 2016, McCloskey *et al.* 2020, Zhang *et al.* 2020, Lliberos *et al.* 2021, Umehara *et al.* 2022, Ben Yaakov *et al.* 2023, Isola *et al.* 2024*a*,*b*, Zhou *et al.* 2024). In the same manner, chronic inflammation can alter the immune cell population in the ovary—both in terms of cell types and the ratio between the different cells. It is thus possible that chronic inflammation at a remote site can elicit a systemic immune response such as cytokine secretion. These cytokines can change the immune cell populations in other organs such as the ovary. The change in immune cells can cause ovarian fibrosis and oocyte attrition and inhibit folliculogenesis, thus causing infertility. This topic is too wide to entirely review in the current manuscript, but it is important to note that this mechanism for affecting female fertility is central to the process.

## Managing chronic inflammation for improved fertility

So far, we have discussed how chronic inflammation can affect female fertility. However, it is possible that outside interventions can reduce inflammation and improve fertility. Another possibility of intervention would be to disconnect the chronic inflammatory condition from its effect on fertility. Either way, outside interventions to reduce the effect of chronic inflammation on females can be developed and some investigation into this subject is reviewed below.

There are several modes of intervention to improve chronic inflammation and enhance fertility. These include preventive medicine (such as lifestyle management through exercise, stress management and good sleep); management of the individual’s environment; addressing microbial factors by improving hygiene; dietary control to manage associated conditions such as obesity; treatment of acute infections before they progress to the chronic stage; and the use of systemic chemical supplements and assisted reproductive technology, including the addition of supplements to IVF media.

Numerous compounds have been employed to mitigate the impact of chronic inflammation on folliculogenesis and female fertility, either through direct exposure to oocytes *in vitro* or via systemic administration (Yang *et al.* 2020*b*). Below are a few notable examples:

*Chitosan oligosaccharide* is a polymer commonly found in fungi and crustaceans, which has been found to possess anti-inflammatory properties and stimulate the immune system. It was shown that chitosan oligosaccharide was able to stimulate the receptors on macrophages to produce IL-1 β and TNF-α and stimulate neutrophils to secrete chemokines such as IL-8, ligands and receptors of other chemokines through the c-Jun/AP-1 signaling pathway (Bahar *et al.* 2012, Liu *et al.* 2013). POI has been identified as a threat to fertility among females of reproductive age and can be attributed to immunological disorders, but chitosan oligosaccharide has been shown to improve POI in a mouse model with minimal side effects and toxicity (Li *et al.* 2023*b*). Moreover, it was shown that chitosan oligosaccharide and its derivatives decrease the production of ROS, lactate dehydrogenase and malondialdehyde as a result of its defense against H_2_O_2_-induced damage by acting as antioxidants and thus are even more likely to produce a positive outcome in preventing the effect of chronic inflammation on female fertility (Liu *et al.* 2009).

*Melatonin* has the potential to activate the release of pro-inflammatory cytokines such as IL-10 and IL-4, and other mediators of the immune system while hampering mechanisms that encourage inflammation, including NF-κB downregulation, SIRT1 activation and Nrf2 upregulation (Yang *et al.* 2020*b*). Melatonin also has the potential to degrade free radicals, including ROS (Yong *et al.* 2021). It has also been shown that melatonin can improve and promote female reproductive processes such as oocyte maturation, increase matured oocyte number, improve fertilization and its rate, and improve the rate and quality of embryonic development, but more research is needed to understand if the immune-modulating activity of melatonin is elicited in each of these responses (Yong *et al.* 2021).

*Resveratrol* is a polyphenol that occurs naturally and is commonly found in certain fruits and nuts. Resveratrol has been proven to have strong antioxidant and anti-inflammatory activities. It has been suggested that the anti-proliferation, anti-inflammatory and anti-tumor effects of resveratrol may be linked to its capacity to mediate the activation of NF-κB initiated by TNF-α. Due to resveratrol’s property of reducing ROS, resveratrol shields oocytes against cytotoxicity which is activated by methyl acetaldehyde (Liu *et al.* 2013). There is mounting evidence that resveratrol elevates the levels of ATP in aged oocytes while reducing levels of chromosome disorders and defects in spindle formation (Itami *et al.* 2015, Ma *et al.* 2015). This makes resveratrol an important potential candidate for improving female fertility, as positive correlations have been drawn between increasing rates of resveratrol and pregnancy rates in mice (Podgrajsek *et al.* 2024).

*Anthocyanins* have been described as multifunctional molecules due to their ability to participate in several biological processes. Anthocyanins have characteristics such as antioxidant, antibacterial and anti-inflammatory. Hence, they can be applied to manage, prevent or treat chronic diseases such as inflammation, metabolic disorders, cardiovascular diseases and cancer (He & Giusti 2010, Valenti *et al.* 2013, Wallace *et al.* 2016).

These interventions are examples of the way we may be able to influence chronic inflammation *in vivo* and *in vitro* to mitigate the effect on female fertility. These types of interventions may ultimately change the way IVF is performed and may work to significantly elevate the overall success rate of IVF.

## Interplay between chronic inflammation, female fertility and aging

Aging is a complex biological process that is impacted by the interaction of many internal processes and external factors. In general, aging causes natural structural deterioration, a decline in function, and a progressive loss of adaptability and resilience. In terms of its effect on the inflammatory system, aging has two major effects that are somewhat contradictory. On the one hand, aging increases the expression and synthesis of pro-inflammatory cytokines by several mechanisms such as the epigenetic alteration of genes implicated in the inflammation process, e.g. the loss of methylation of the TNF promoter, a process termed inflammaging (Bjornsson *et al.* 2008). Inflammaging, an age-related increase in pro-inflammatory markers in blood and tissues, is a powerful risk factor for several illnesses that are quite common and frequently cause disability in the elderly population (Ferrucci & Fabbri 2018). On the other hand, aging is also linked to progressive changes in immunological reactivity to acute infections, often known as immunosenescence. It entails a decrease in neutrophil and macrophage activity, eventually leading to a reduction in the ability to combat pathogens as age increases (Gomez *et al.* 2005). Therefore, it is impossible to accurately predict the effect of aging on immune function in a specific system or organ, and this needs to be empirically tested.

Female fertility sharply decreases with age and is considered the first organismal system to deteriorate with aging. Female reproductive aging causes reduced oocyte quality, fertility loss, irregular menstrual cycles and in humans—eventually menopause. Inflammatory processes seem to play an important role in ovarian aging. It has been shown that a pro-inflammatory microenvironment can be linked to ovarian aging in mice (Lliberos *et al.* 2021). It has also been demonstrated that older ovaries had higher levels of inflammatory cytokine gene transcription, where TNF-α, IL-6, IL-10 and IL-18 showed significantly increased levels (Zhang *et al.* 2020). The depletion of ovarian reserve and the decreased sensitivity of gonadotropin-releasing hormone, which results in a dysregulation of estrogen signaling, are both thought to contribute to the age-associated decline in fertility. It is believed that these changes at a specific stage of reproductive age contribute to altering macrophage functions in ovaries, favoring the development of fibrosis and a pro-inflammatory condition because estrogen signaling affects macrophages' production of estrogen receptors (Zhang *et al.* 2020). Studies in bovine models have demonstrated that oviduct epithelial cells undergo age-related changes, which are primarily characterized by the development of an inflammatory milieu. Older animals in bovine models had endometrial cells that spontaneously displayed increased amounts of DNA damage, IFN signaling and inflammatory signaling (Tanikawa *et al.* 2017). Thus, inflammation and its associated factors contribute to the deterioration of oocyte quality in the aging process through various mechanisms as discussed above. Therefore, due to the inherent connection between inflammatory processes and aging, it could be claimed that similar approaches to interventions should be taken in both cases. This calls for the administration of anti-inflammatory agents during reproductive aging and anti-aging agents in chronic inflammation. This direction could become a promising avenue to combat female infertility, which is affected both by inflammation and aging.

## Conclusion

**Figure 1:**
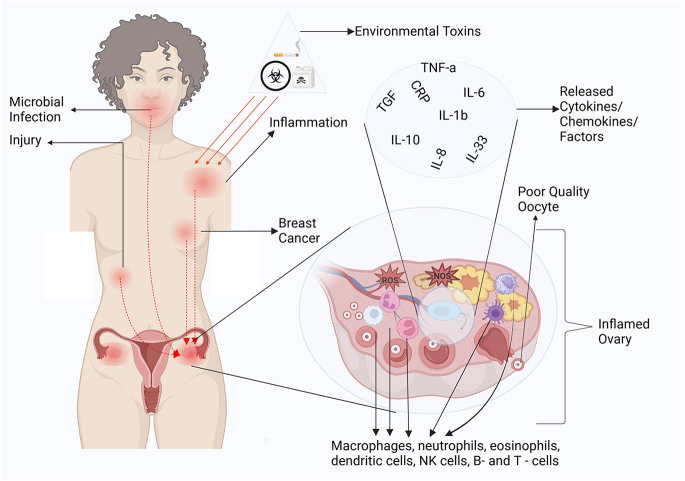
Schematic representation of the impact of systemic inflammation on ovarian function. Environmental toxins, microbial infections and injuries trigger inflammation, leading to the release of pro-inflammatory cytokines, chemokines and factors such as TNF-α, IL-6, IL-1β, IL-8, IL-33, IL-10 and TGF-β. These inflammatory signals contribute to oxidative stress (ROS) within the ovarian microenvironment, resulting in poor-quality oocytes and follicular dysfunction. Immune cells, including macrophages, neutrophils, eosinophils, dendritic cells, natural killer (NK) cells and B and T cells, infiltrate the inflamed ovary, exacerbating damage. The interconnected pathways between systemic inflammation, breast cancer and reproductive health highlight the far-reaching effects of chronic inflammatory states on female fertility.

It is clear from existing data that chronic inflammation strongly affects female fertility at many levels and through several possible mechanisms (see Fig. 1). However, the full details of the mechanisms underlying the impact of chronic inflammation on female fertility are currently not understood completely. There is more need to investigate the underlying mechanisms and details of: i) how chronic inflammation from distant parts of the body can get to the ovary and impair fertility; ii) the combinatorial effect of multiple factors such as genetic, environmental and dietary factors that affect reproductive health.

This will help to discover new drugs to fight against infertility. Moreover, the connections between chronic inflammation and reproductive aging should be further investigated, as this could lead to the discovery of new druggable pathways.

## Declaration of interest

The authors declare that there is no conflict of interest that could be perceived as prejudicing the impartiality of the work reported.

## Funding

This research received funding from Merck (a personal grant to MK).

## Author contribution statement

MK conceived and wrote the paper. SA participated in the writing of the paper.
